# Work & life stress experienced by professional workers during the pandemic: a gender-based analysis

**DOI:** 10.1186/s12889-024-18677-6

**Published:** 2024-05-29

**Authors:** Melissa Corrente, Jungwee Park, Henrietta Akuamoah-Boateng, Jelena Atanackovic, Ivy Lynn Bourgeault

**Affiliations:** 1 School of Sociological and Anthropological Studies, University of Ottawa, 120 University Private, 75 Laurier Ave E, Ottawa, ON K1N6N5 Canada; 2 Health Analysis Division, Statistics Canada, 150 Tunney’s Pasture Driveway, Ottawa, ON Canada

**Keywords:** Work stress, Life stress, Professional workers, Gender-based analysis

## Abstract

The COVID-19 pandemic impacted work and home life exacerbating pre-existing stressors and introducing new ones. These impacts were notably gendered. In this paper, we explore the different work and home life related stressors of professional workers specifically as a result of the COVID-19 pandemic through the gender-based analysis of two pan Canadian surveys: The Canadian Community Health Survey (2019, 2020, 2021) and the Healthy Professional Worker Survey (2021). Analyses revealed high rates of work stress among professional workers compared to other workers and this was particularly notable for women. Work overload emerged as the most frequently selected source of work stress, followed by digital stress, poor work relations, and uncertainty. Similar trends were noted in life stress among professional workers, particularly women. Time pressure consistently stood out as the primary source of non-work stress, caring for children and physical and mental health conditions. These findings can help to develop more targeted and appropriate workplace mental health promotion initiatives that are applicable to professional workers taking gender more fully into consideration.

## Introduction

The COVID-19 pandemic impacted many aspects of everyday life, including work and home life exacerbating pre-existing stressors and introducing new ones. Adverse working conditions often lead to work stress [1–3] along with psychological distress and other mental health issues among employees [4, 5]. Many professional workers were part of the frontline response to the pandemic which entails heightened stress. Other professional workers shifted to remote work and telecommuting which contributed to other forms of work-related stress [6]. Among all professional workers, increased workload, lack of control, and the uncertainty of the pandemic have contributed to higher levels of stress.

Blurred boundaries between work and home life has also been significantly impacted by the pandemic, especially for those ‘working from home’ [7]. This blurring of boundaries led to increased workload and longer working hours, particularly for women [8], further contributing to stress. Virtual working increases employees’ workload and enables work to interfere more frequently with personal or family life [9–11]. Some professionals felt isolated and disconnected from their colleagues and the work environment during the pandemic, leading to higher levels of depression and anxiety [12–14]. The pandemic highlighted the need for connection among professional workers so employees feel less isolated when working from a remote or hybrid setting.

Gender has emerged as having a significant impact on the changing dynamics of both the workplace and home life for all professionals during the COVID-19 pandemic and in turn on work and life stress [15, 16]. Taking the impact of gender more fully into consideration in understanding these changing dynamics can help to develop more targeted and appropriate workplace mental health promotion initiatives for professional workers.

### Purpose

In this paper, we explore the different work and home life related stressors of professional workers as a result of the COVID-19 pandemic through an explicit gender-based analysis of two pan Canadian surveys. Our gender-based analysis included a focus on gender identity as well as a more nuanced approach that included gendered roles with respect to non-work stress and the gendered nature of the professions studied.

### Literature review

#### Work stress among different professional workers

Stress impacts work practices under normal conditions [17–19]. In many studies, working long hours is frequently mentioned as a source of work stress among both health [20–22] and non-health professionals [23, 24]. Some of the mental health challenges facing health professionals are connected to the inflexible and irregular work schedules which often include shift work and required overtime [25, 26]. For non-health professionals like teachers, mental health challenges are associated with work overload, multiple demands, emotional labour and a lack of psychological safety [27].

Early on in the pandemic, the primary source of work stress among health professionals, for example, was the diversity and quantity of information from diverse sources [3]. Health professionals in general felt isolated because they were not involved in the care organization’s decision-making process before and during the first wave of COVID-19. They found the uncertainty about when the pandemic would be under control extremely stressful [28]. Working with COVID-19 patients placed health professionals at a greater risk of experiencing higher levels of stress, anxiety and depression [29–31]. Taking protective measures (e.g. washing hands, wearing a mask, taking own temperature, etc.) was the coping strategy used most frequently by health and non-health professionals [32]. The effects of work stress on mental health during the pandemic may be felt for years [33, 34].

For non-health professionals, COVID-19 caused a drastic change. For teaching professionals, for example, it eliminated some protective factors for managing stress and mental health at work, such as social support [35]. Teachers rely on social support to help mitigate the negative effect of workplace stress [36, 37], but virtual teaching changed the dynamic of the teaching profession [38]. According to Mental Health Research Canada (2020), the number of teachers reporting high levels of anxiety increased 500% since the pandemic. Similarly, academic professionals reported difficulties in technical aspects and the absence of “face-to-face” eye contact with university students [39, 40]. Teamwork and feeling appreciated at work were noted as protective factors leading to lower odds of stress, anxiety, and job burnout and these were notably absent in remote work [20, 41, 42]. Overall, work related factors seem to be much stronger predictors of outcomes such as stress and burnout in comparison to individual factors [43].

#### Life stress among professional workers

The elusive work-life balance became even more difficult to achieve during the pandemic. Working hours directly affect the work-life balance of professional workers. As working hours of health professionals increased, work-life balance decreased [44, 45]. Boundaries that traditionally separated work and home life became blurred for many workers [46]. Professional workers from all different areas were dissatisfied with their work-life balance during the pandemic [41, 47–49]. Humphries et al. [50] found that 73% of hospital-based medical professionals were feeling the strain of work-life imbalance, which negatively affected their lives and well-being.

Research reported that the stress, anxiety, and burnout of health professionals caring for COVID-19 patients affected their quality of life [51–55]. Anxiety levels of healthcare workers who had children were found to be higher than those who did not have children [51, 56]. During the pandemic many non-healthcare professionals such as teachers and accountants had their children at home or found it difficult to find childcare which only increased their stress levels [57, 58]. Professions where women workers predominate, midwives, teachers, and nurses, all reported poor work-life balance and mentioned it as a key source of stress [41, 47–49].

#### Gender differences in work and life stress

Work and life stress are deeply influenced by gender. Workplace mental health studies reveal poorer mental health among women [59–61]. For instance, some studies find that women report significantly high levels of emotional exhaustion compared to men [21, 62] along with higher work-related stress and anxiety [6, 22]. A discussion surrounding gender differences in mental health at work cannot ignore the gendered division of labour in the home environment. Women report higher levels of stress providing for dependents [6, 23, 63]. One study found that job strain has a direct adverse effect on life stress among women but not among men [64].

Gender has been noted as one of the main predictors of early burnout that mainly affects women healthcare workers who are more likely to develop work-related stress [65, 66]. For non-health professionals, although women are still more predominant in the teaching profession, for example than men [67], the double burden of care work that exists for teachers who are also mothers has only increased throughout the pandemic [27]. Research among accounting professionals show that women were more likely to experience mental distress while working from home than men [57]. Interestingly, male accountants with children at home experienced increased well-being, but reported needing more time to recover after a day’s work.

Gender differences at work can be connected to factors such as inequitable distribution of working and employment conditions, along with gender-based harassment and bullying [59, 68–72]. Huang et al. [66] found a strong relationship between the number of working hours and occupational burnout in women professionals even when variables such as age, marital and parental status and household responsibilities are controlled. One study investigating the gendered nature of work, stress, and mental health found that women professionals reported higher levels of psychological demands and had higher rates of work absences than non-professional women workers [73].

Women who work in health professions face an increased workload due to the increased number of patients with COVID-19 [54]. High levels of depression and anxiety were more common among women health professionals in China [52]. Research from Portugal reports that burnout levels among women health professionals were over four points higher on average in comparison with men [74].

In sum, there are notable gender differences found in work and non-work related stress among professional workers but equally notable research gaps in the experience and sources of work and life stress, especially from a comparative perspective and further which takes into consideration the impact of the pandemic on these differences.

## Methods

This paper undertakes a secondary data analysis of two different pan Canadian surveys to address the gendered nature of the pandemic impact on professional workers: The Canadian Community Health Survey (2019, 2020, 2021) administered by Statistics Canada and the Healthy Professional Worker Survey (2021) undertaken by a pan Canadian research team. Across the two datasets, we focused on the following professional worker–—academics, accountants, dentists, nurses, physicians and teacher–—which represent a range of work settings and gender composition. Utilizing the StatCan specific datasets also allows us to compare the circumstances of these professional workers with non-professional workers.^1^

### Canadian community health survey

#### Data source

This study used the annual cycles of the Canadian Community Health Survey (CCHS). The CCHS is a cross-sectional survey that collects information related to health status, health care utilization, and health determinants for the Canadian population. This analysis focused on the data on self-reported mental health outcomes 1) before the pandemic (CCHS 2019 annual data) and 2) since the pandemic using the two cycles: CCHS 2020^2^ – September to December 2020, and CCHS 2021. The two data cycles (2020, 2021) since the pandemic were combined and analyzed to attain sample sizes large enough to yield reasonable estimates. The combined data were weighted and adjusted by a factor of two to represent the Canadian household population as two cycles were combined [76, 77]. The combined estimates do not represent the population of any particular year; rather they reflect the average Canadian household population across the 2020 (September) to 2021 period [78].

#### Sample

The sample size of the combined data (CCHS 2020 -September to December 2020 and CCHS 2021) was 32,214 participants (15,626 men and 16,588 women) representing 18,538,964 persons aged 15 or older, who reported “worked at a job/business last week” or “absent from work/business last week.” Among them, the sample of Case Study Professional (CSP) workers were 2,533 representing 1,420,302 workers (490,502 men and 929,800 women) in 2020 and 2021. The response rates for the cycles were: 23.6% (2020), and 24.1% (2021) respectively. The sample size of workers aged 15 or older in the CCHS 2019 was 28,616 representing 18,632,424 workers in 2019. Among them, the sample of CSP workers were 2,221 representing 1,450,294 (471,951 men and 978,343 women) in 2019. The response rate for the 2019 CCHS was 54.4%.

#### Measures

CSP workers were identified based on self-reported occupations translated to the 4-digit codes from the National Occupational Classification for Statistics (NOC) 2016 from the CCHS 2019 and CCHS 2020–2021 data. CSP workers included accountants, nurses, doctors, dentists, professors, and teachers.

- Self-perceived life stress measures an individual’s perception of overall stress in life. Respondents were asked, “Thinking about the amount of stress in your life, would you say that most days are: not at all stressful? not very stressful? a bit stressful? quite a bit stressful? extremely stressful?” Respondents answering quite a bit or extremely stressful were classified as having high self-perceived life stress.
- Self-perceived work stress at the main job or business in the past 12 months was measured by asking: “Would you say that most days at work were: not at all stressful? not very stressful? a bit stressful? quite a bit stressful? extremely stressful?” Respondents answering quite a bit or extremely stressful were classified as having high self-perceived work stress.

#### Analytical techniques

Descriptive statistics analyses were conducted to provide prevalence rates of self-perceived life stress and self-perceived work stress. Multivariate logistic regression analyses were conducted to examine the effects of occupations on self-perceived life and work stress. Adjusted odds ratios of high life stress and work stress for all, men, and women workers were presented. Age, sex, and type of work (full-time vs. part-time, essential vs non-essential) were controlled. Statistical significance was indicated based on the tests with a *p*-value of less than 0.05. Bootstrap weights were used for significance tests.

### Healthy professional worker partnership

#### Data source

As part of the Healthy Professional Worker Partnership, a bilingual (French–English) self-administered survey was launched across Canada to understand the intersectional and contextualized experiences of professional workers during the COVID-19 pandemic. The survey was made available online through the Qualtrics platform, and recruitment took place between the end of November 2020 and early May 2021. A convenience sampling approach was employed, utilizing professional association partner organizations, direct email invitations, and social media for recruitment. Research Ethics Board approval was obtained from the University of Ottawa and 16 other collaborating universities.

The survey design included common questions related to the pandemic impact applicable to all participants. Participants were then guided to relevant questions based on their initial profession-specific response, employing a skip-logic feature. The survey took approximately 20 min to complete, with only the initial question being mandatory.

#### Sample

Data analysis was conducted on surveys with a completion rate of 90% or higher, resulting in 3369 retained surveys across the following case studies: Academia (379; *(250 women/92 men)*), Accounting (312; *(202 women/94 men)*), Dentistry (397; *(194 women/185 men)*), Medicine (310; *(258 women/46 men)*), Midwifery (202; *(188 women/0 men)*), Nursing (1013; *(929 women/60 men)*), and Teaching^3^ (756; *(585 women/140 men)*). Overall, 2606 women, 617 men, 52 respondents identified as gender fluid, preferred to self-describe or preferred not to answer and 94 people did not respond to the gender question. The calculation of the response rate was precluded as a result of employing a convenience sampling approach.

#### Measures

Gender was identified from the question, “*What is your gender?*” where it was noted that “Gender refers to the gender that a person internally feels ‘('gender identity' along the gender spectrum) and/or the gender a person publicly expresses ‘('gender expression') in their daily life, including at work, while shopping or accessing other services, in their housing environment or in the broader community. A person's current gender may differ from the sex a person was assigned at birth (man or woman) and may differ from what is indicated on their current legal documents. A person's gender may change over time.” The response categories included 1) Woman 2) Man 3) Non-binary/Gender fluid and 4) Prefer to self-describe.

Profession was determined in response to the question, “*What is your primary profession/professional role?*”.

The following outcome variables were asked first of respondents to reflect the present context where they were [during the pandemic] and in February of 2020 [prior to the pandemic]:

- Ratings of life stress were identified through the following question, “Thinking about the amount of stress in your life in general (excluding work-related stress) since the start of the COVID-19 pandemic, would you say that most days are: not stressful at all? not very stressful? a bit stressful? quite stressful, extremely stressful?”
- Sources of life stress were identified through the following question, “Thinking about stress in your life in general (excluding work-related stress) since the start of the COVID-19 pandemic, what would you say contributes to the feelings of stress you may have? (select all that apply) physical health problem or condition, emotional or mental health problem or condition, personal safety, debt/financial situation, discrimination, caring for children, caring for others (outside of work), time pressure/not enough time, family safety, family health condition (critical or chronic disease), grief/loss of family member, marital or relationship challenges, intimate partner violence, other (please specify), not applicable.”
- Ratings of work stress were identified through the following question, “Thinking about the amount of stress in your work life since the start of the COVID-19 pandemic, would you say that most days are, not stressful at all? not very stressful? a bit stressful? quite stressful? extremely stressful?”
- Sources of work stress were identified through the following question, “Since the start of the COVID-19 pandemic, which of the following sources of work stress are most relevant to you? (select all that apply), physical safety, including exposure to occupational hazards, work overload, multiple demands and deadlines, “digital stress” (i.e. emails, online forms, Electronic Medical Records), no control over work hours or no flexibility in schedule, lack of autonomy, critical events and/or incidents in the workplace, ethical dilemmas, employment insecurity, stress of running a practice, managing people, meeting budgets, risk of lawsuits and risk to reputation, lack of psychological safety at work including bullying, harassment, discrimination or workplace violence, poor relations with management or administration and feeling shut out of decision making, poor relations with immediate supervisor, poor relations with co-workers/colleagues, poor relations with students, other (please specify).”

#### Analytic techniques

Various analysis methods were employed on non-missing values, including cross-tabulation, mean testing, regression, and chi-square tests of association. Significance was determined using a chi-square test of association with a significance level of less than 0.05. Initially, a cross-tabulation with a chi-square test of association was performed to analyze all survey questions in relation to the main outcome variables. Additionally, a test of equality of two proportions was used to examine significant differences in experiences between the case study populations as well as among different genders.

### Findings

We begin with a presentation of the work stress findings from the Canadian Community Health Survey (CCHS) analysis that enable a comparison with non-professional workers followed by the profession focused analysis of the Healthy Professional Worker (HPW) study which enabled a deeper dive into the sources of stress. Findings for non-work stress from both sources are explored subsequently.

### Work stress (CCHS)

**Fig. 1:**
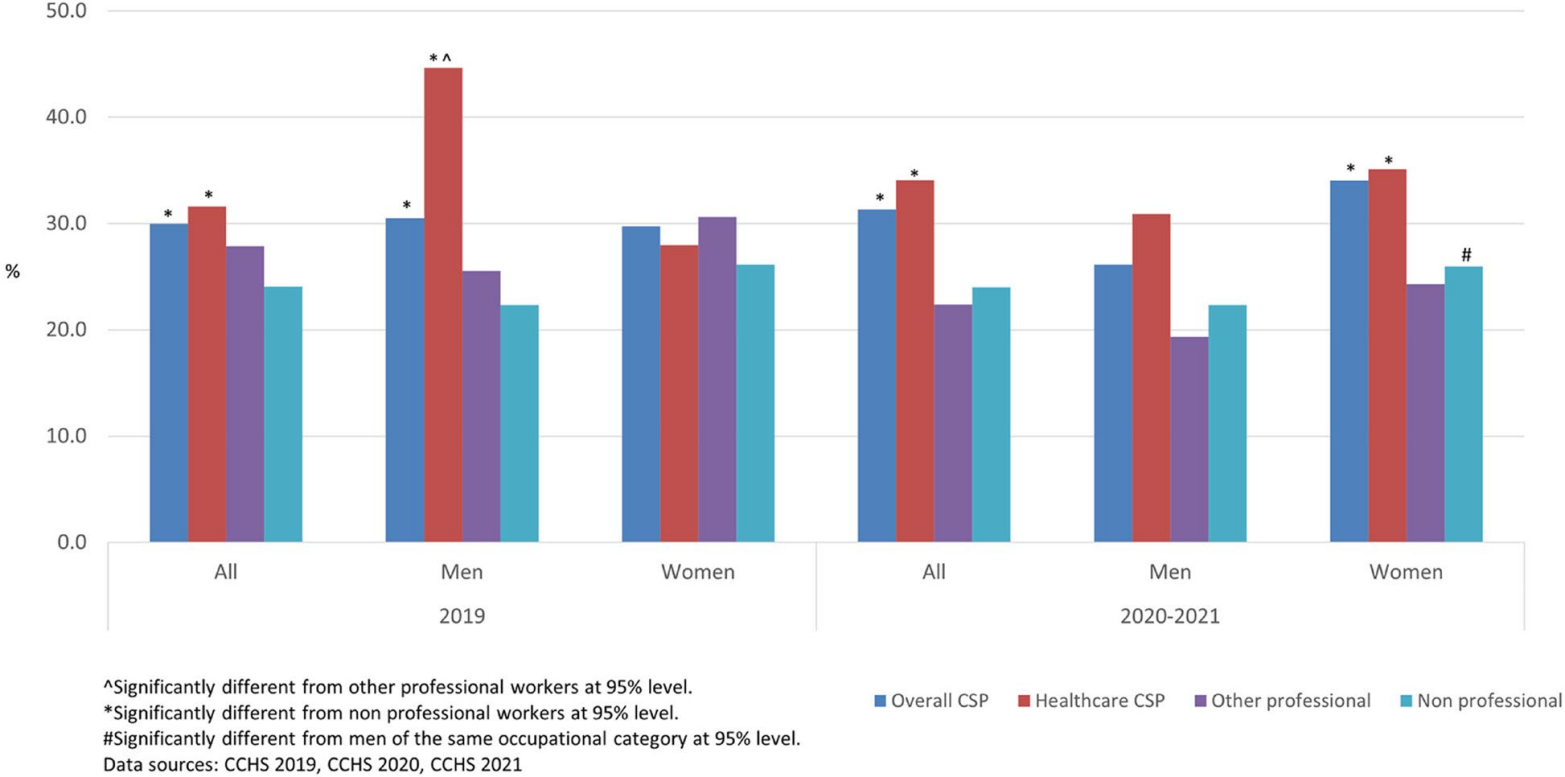
Rate (%) of high self-reported work stress (quite a bit or extremely stressful) life stress by occupation, 2019, 2020–2021, Canada

In both 2019 and 2020–2021, the higher rates of (quite a bit or extremely stressful) work stress were found among CSP workers compared to other workers (Fig. 1). Health professionals were consistently more likely to report high work stress than other workers. The rate of high work stress among men health professionals in 2020–2021 slightly increased to 35% from 33% in 2019 whereas more women health professionals reported high work stress during the pandemic in 2020–2021 compared to 2019 (from 48 to 61%). During the pandemic, women CSP workers were more likely to report high work stress compared to non-professional workers and other professional workers. As well, women CSP workers and women health professionals were more likely to report high work stress compared to men. In 2020–2021, about half of women CSP workers reported high work stress compared to about one third of men CSP workers. The proportion of women health professionals reporting high work stress was about 20 percentage points higher than that of men (61% vs 41%).

**Fig. 2:**
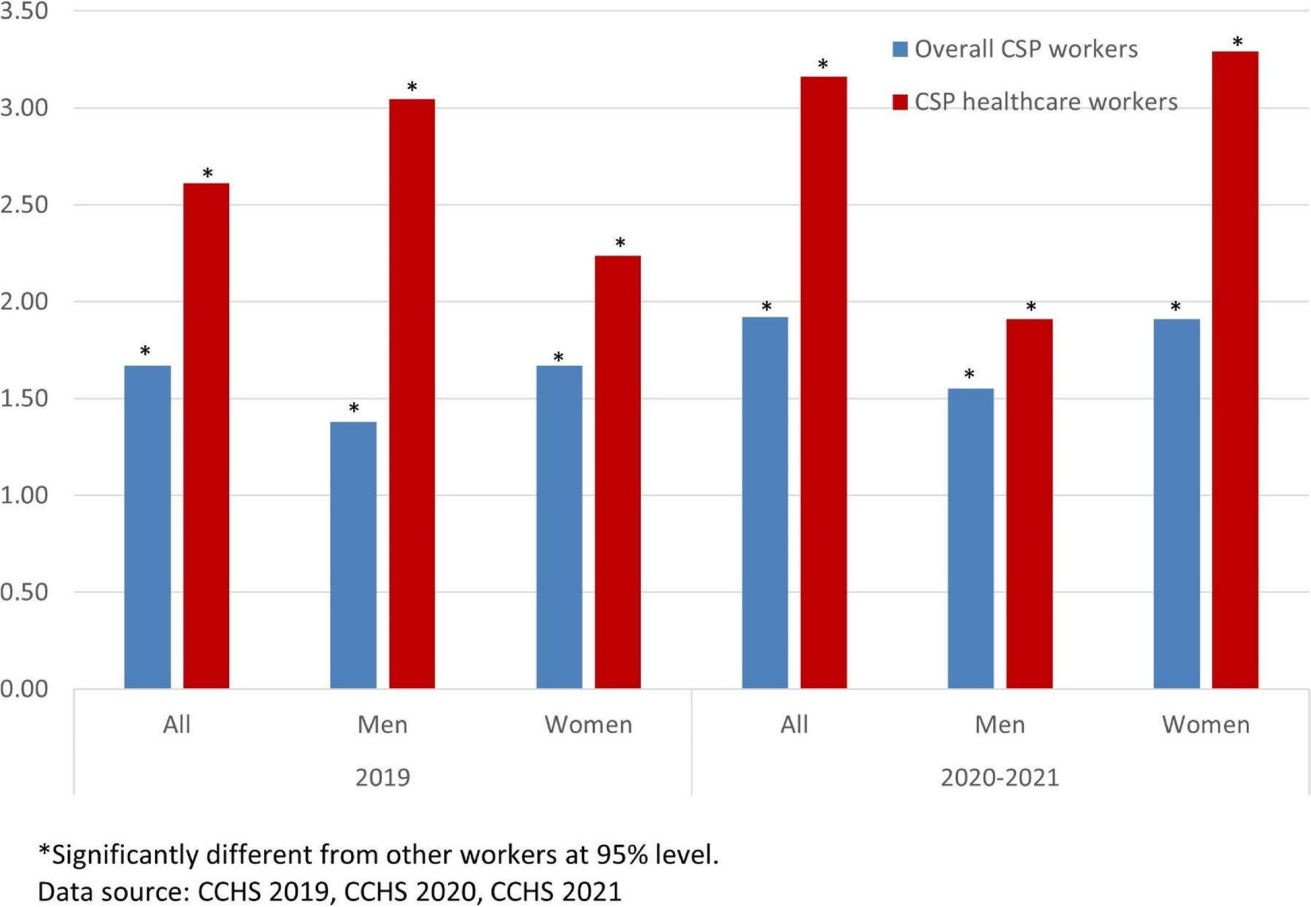
CSP workers’ odds ratios for high self-reported work stress compared to other workers

Figure 2 shows that during the pandemic the odds for women CSP workers to report high work stress were greater compared to other workers. Especially, the odds for women doctors and nurses during the pandemic were about 3.3 times greater than that for other workers. Women CSP workers were twice as likely to report high work stress as non-CSP workers. These odds for women in 2020–2021 showed considerable increases from 2019. The odds for men health professionals to report high work stress compared to other workers decreased during the pandemic period, from 3.1 in 2019 to 1.9 in 2020–2021.

### Work stress and its sources (HPW)

The HPW survey assessed participants' self-reported work stress on a scale from 1 to 5. All professions reported higher levels of work-related stress during the COVID-19 pandemic compared to the pre-pandemic period. Gender disparities were noted in the alteration of work-related stress, with women indicating a significantly higher average increase in work stress during the COVID-19 pandemic in comparison to their stress levels before it (0.60 for women compared to 0.48 for men). A comparable gender disparity was noticed in academia, where women reported a difference of 0.43, while men reported 0.16. Significant gender differences were not detected in other professions.

**Fig. 3:**
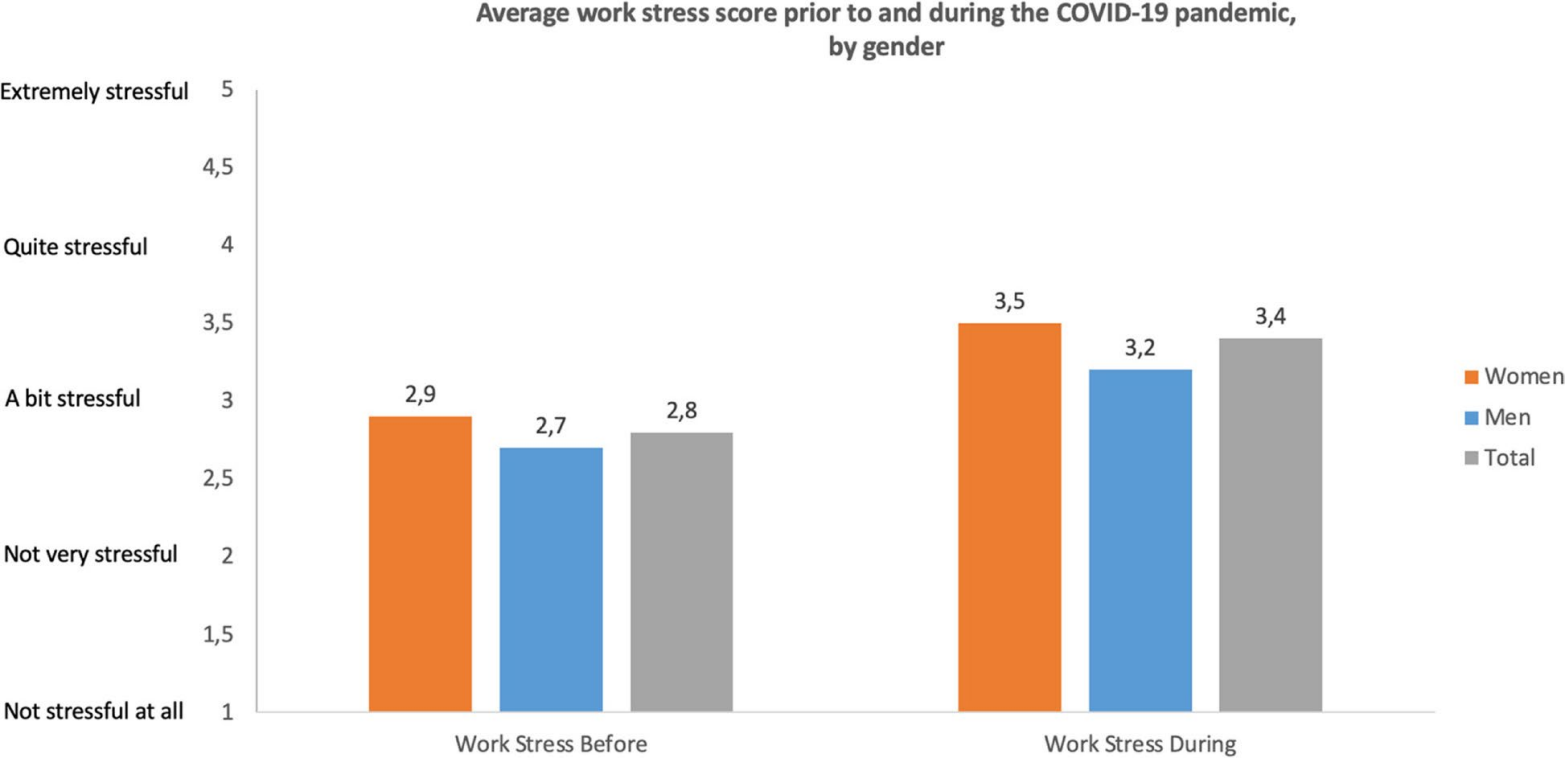
Self-reported work-related stress before and during the COVID-19 pandemic

Prior to COVID-19, the average stress score for all professionals was 2.8, indicating a slightly stressful environment (Fig. 3). During the pandemic, scores were significantly higher at 3.4, which falls between “a bit stressful" and “quite stressful”." This trend was consistent among both women (2.9 vs. 3.5) and men (2.7 vs. 3.2). Although both women and men experienced a notable increase in stress levels during the COVID-19 pandemic, women reported higher mean scores both before and during the pandemic compared to men.

Across the professional dataset (academics, accountants, dentists, nurses, physicians and teachers), work overload emerged as the most frequently selected source of work stress, except in dentistry where it ranked third. Digital stress, poor work relations, and uncertainty were also prominently cited as top sources of work stress across various professions. Furthermore, feelings of exclusion from decision-making processes were also reported as stress-inducing factors for those in academia, midwifery, and teaching.

**Fig. 4:**
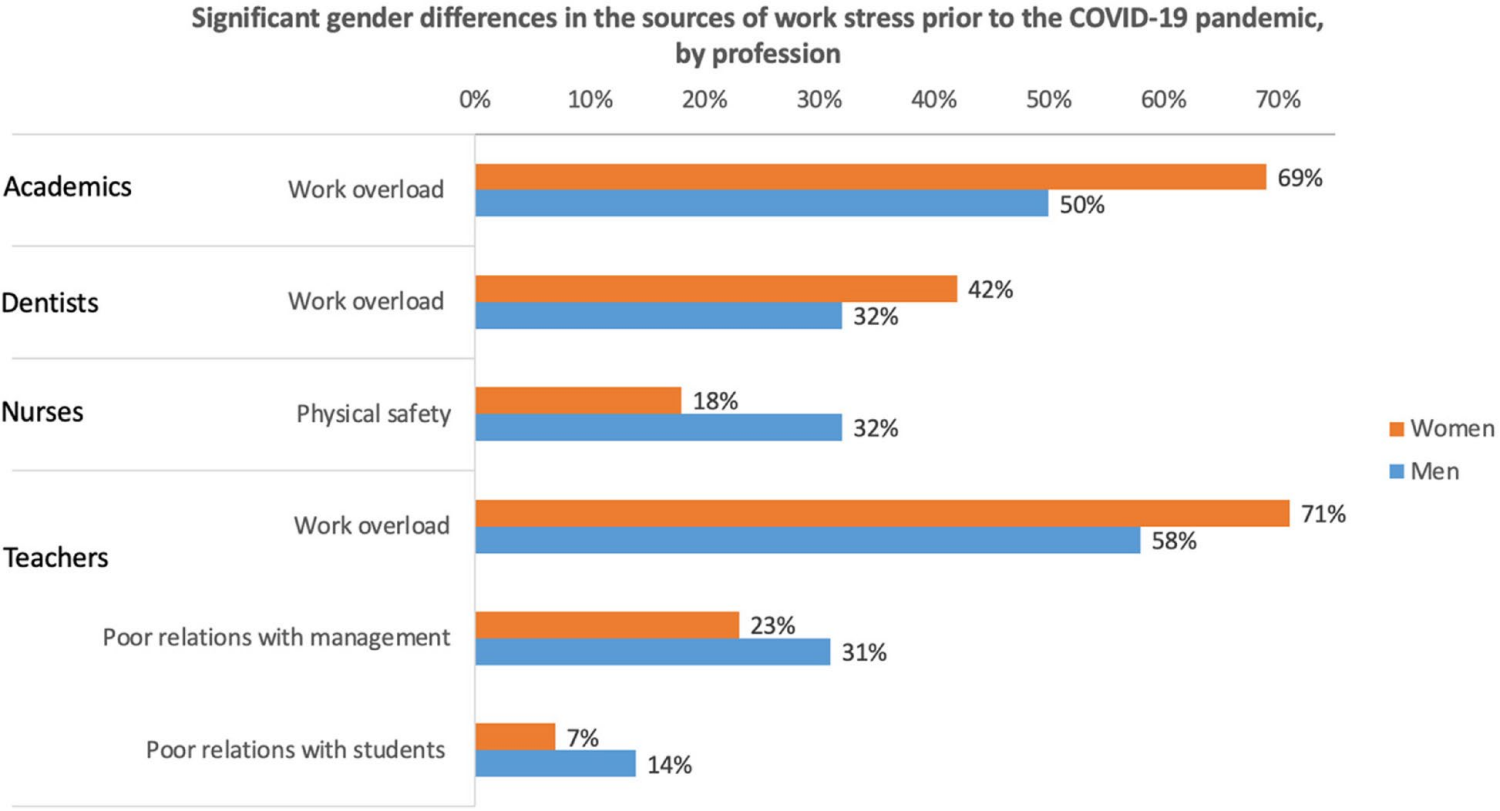
Significant gender differences in the sources of work stress prior to the COVID-19 pandemic by profession

**Fig. 5:**
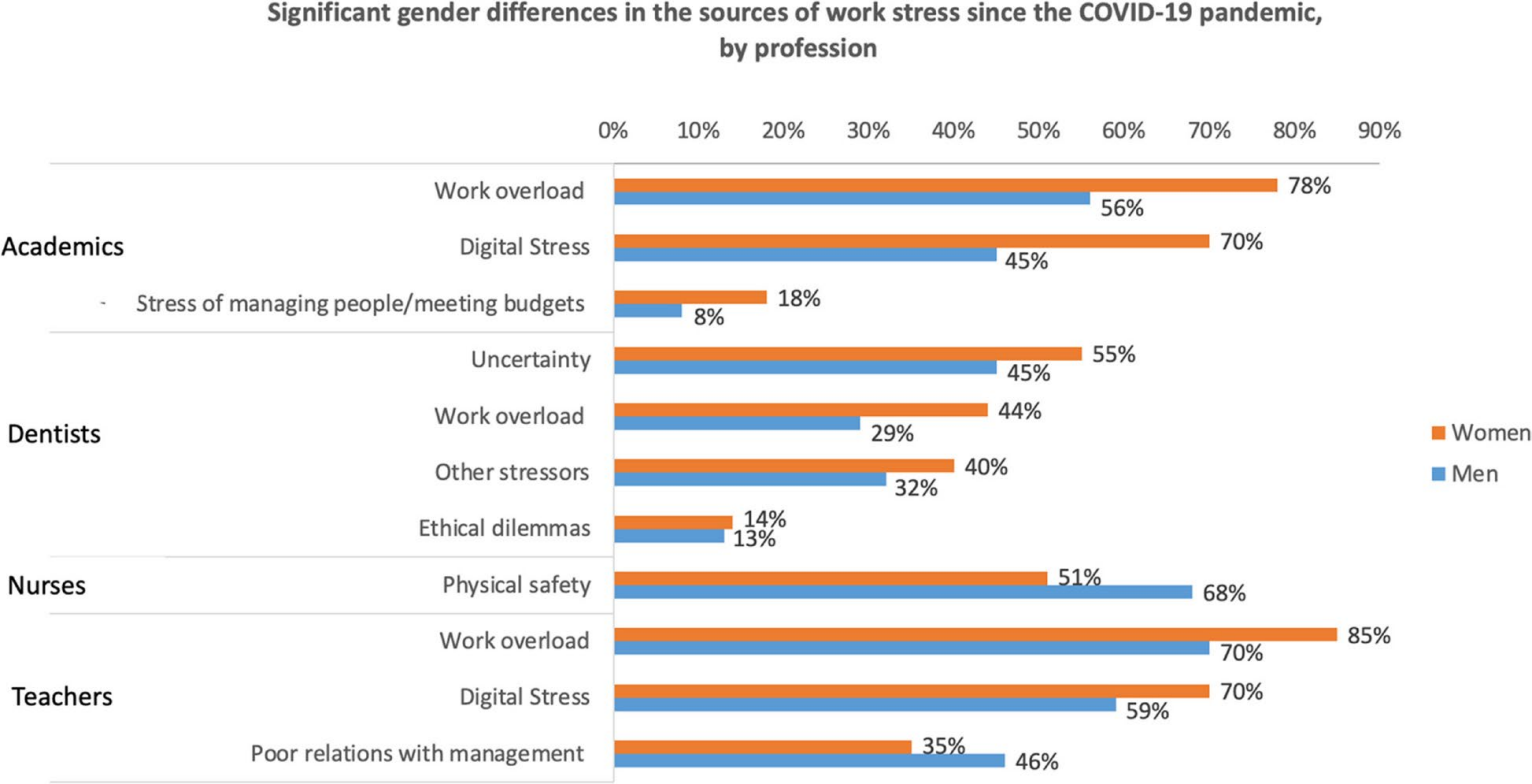
Significant gender differences in the sources of work since the COVID-19 pandemic by profession

Unique stressors specific to certain professions were identified by dentistry and nursing respondents. For example, dentistry professionals highlighted stress related to managing a practice and coping with uncertainties. Nursing professionals, on the other hand, faced additional stressors such as concerns for physical safety and ethical dilemmas. Each of these stressors highlights the distinct stress profiles associated with different professions during the pandemic (Figs. 4 and 5).

Notable gender disparities were evident in the selection of work overload in academia, both before (69% women vs. 50% men) and during COVID-19 (78% women vs. 58% men). During the pandemic, higher proportions of both men and women reported this stressor.

While significant gender differences were not initially observed in the selection of other stressors before COVID-19, during the pandemic, gender disparities became apparent in multiple areas, including stress related to practice management, people management, and budgeting (18% women vs. 8% men), as well as digital stress (70% women vs. 45% men).

Dentists displayed significant gender differences in work overload, both before (42% women vs. 32% men) and during COVID-19 (44% women vs. 29% men). During the pandemic, gender differences emerged in uncertainty (55% women vs. 45% men), ethical dilemmas (14% women vs. 13% men), and other stressors (40% women vs. 32% men).

Clear gender disparities were noticeable among teachers. These disparities encompassed challenges such as poor relations with management or administration and feeling shut out of decision-making, both before (23% women vs. 31% men) and during the COVID-19 pandemic (35% women vs. 46% men). Additionally, significant gender differences emerged in the reporting of issues like work overload, facing multiple demands, and meeting deadlines, both before (71% women vs. 58% men) and during the pandemic (85% women vs. 70% men). Prior to COVID-19, a disparity was evident in poor relations with students (7% women vs. 14% men), while during the pandemic, digital stress exhibited significant gender differences (70% women vs. 59% men).’t's worth noting that higher proportions of these challenges were not exclusively associated with women in the teaching profession.

Among nurses, the sole work-related stressor with significant gender differences was physical safety, including exposure to occupational hazards, before (18% women vs. 32% men) and during COVID-19 (51% women vs. 68% men), with men reporting higher proportions.

No gender differences were notable in accounting. Midwifery was excluded from the gender analysis due to the absence of male participants.

### Life stress (CCHS)

**Fig. 6:**
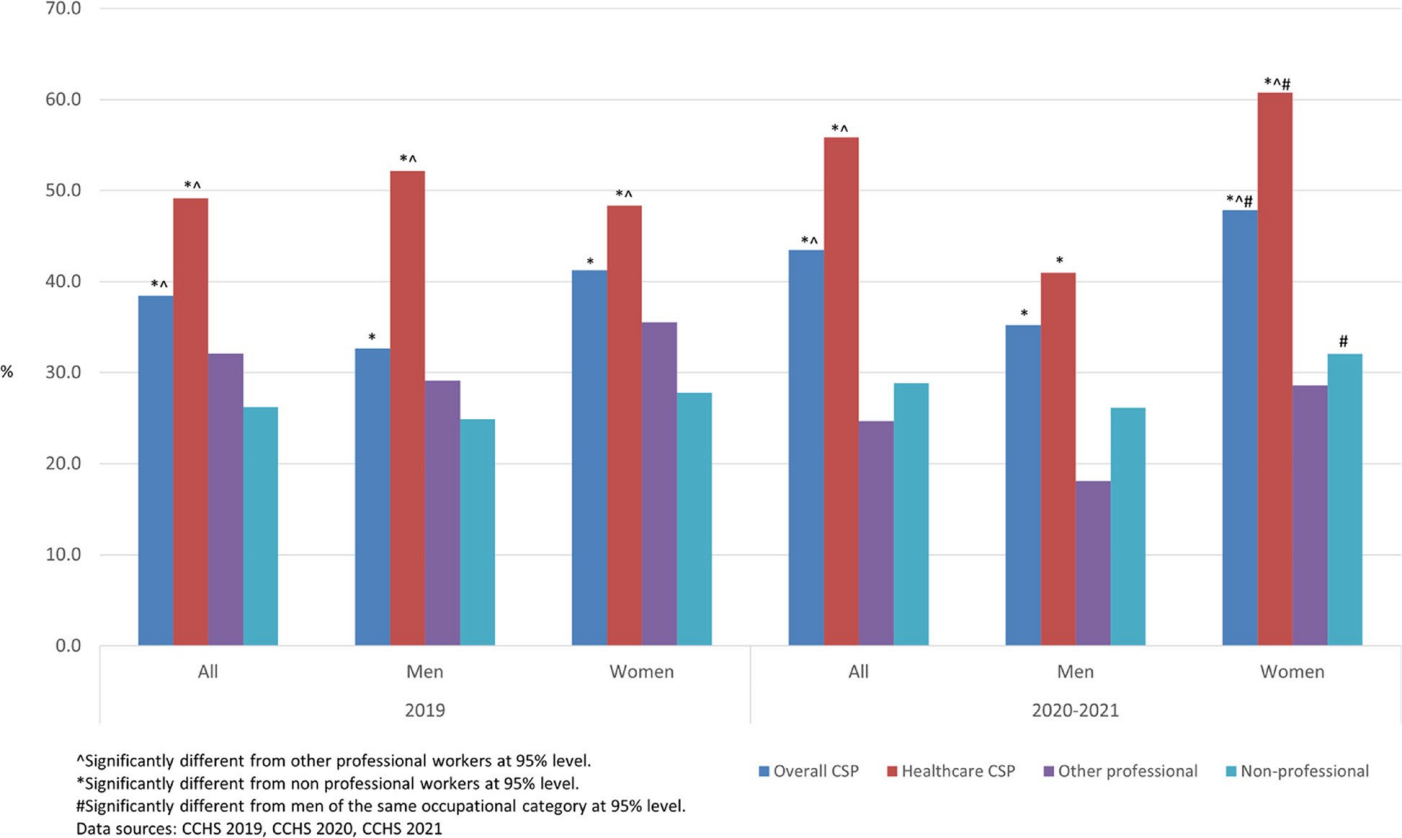
Rate (%) of high self-reported life stress (quite a bit or extremely stressful) life stress by occupation, 2019, 2020–2021, Canada

Figure 6 shows that overall CSP workers and health professional workers (doctors and nurses) were more likely to report high (quite a bit or extremely stressful) life stress compared to non-professional workers. During the pandemic, women CSP workers were more likely to report high life stress compared to their non-professional counterparts. Men CSP workers were not significantly different in life stress compared to other workers in 2020–2021. It was different from before the pandemic (2019) when men CSP workers were more likely to report high life stress than non-professional workers, and men health professional workers were more likely to report high life stress than other workers.

**Fig. 7:**
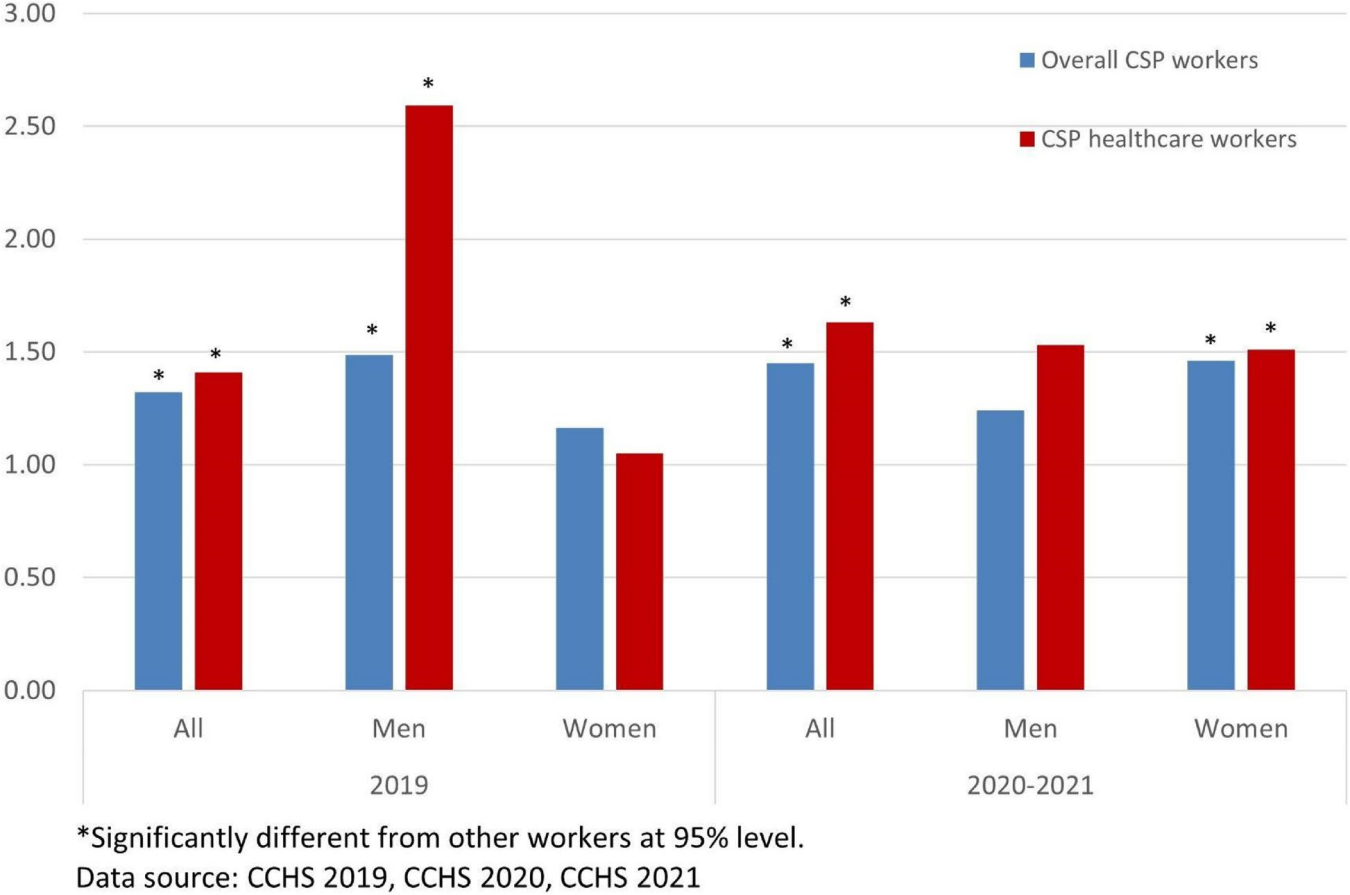
CSP workers’ odds ratios for high self-reported life stress compared to other workers

In 2019, the odds for male CSP workers and men health professional workers to report high life stress were significantly higher compared to that for other workers (Fig. 7). Especially, men health professional workers were more than 2.5 times as likely to report high life stress as other men workers. The odds for women CSP workers were not statistically different from that for other women workers in 2019. In 2020–2021, during the pandemic, however, the odds for women CSP workers and women health professional workers to report high life stress were significantly higher (about 1.5 times) than that for other workers. During the pandemic, the odds for men CSP workers were not statistically different from that for other men workers.

### Life stress and its sources (HPW)

The HPW survey provided participants with the opportunity to assess their stress levels in their personal lives. Analysis of the HPW data revealed higher life (or non-work-related) stress among the survey participants during the COVID-19 pandemic compared to the pre-pandemic period. The overall population reported a mean stress score of 2.4, indicating a proximity to "not very stressful" before COVID-19. This measure was 3.0 during the pandemic, leaning closer to "a bit stressful".

**Fig. 8:**
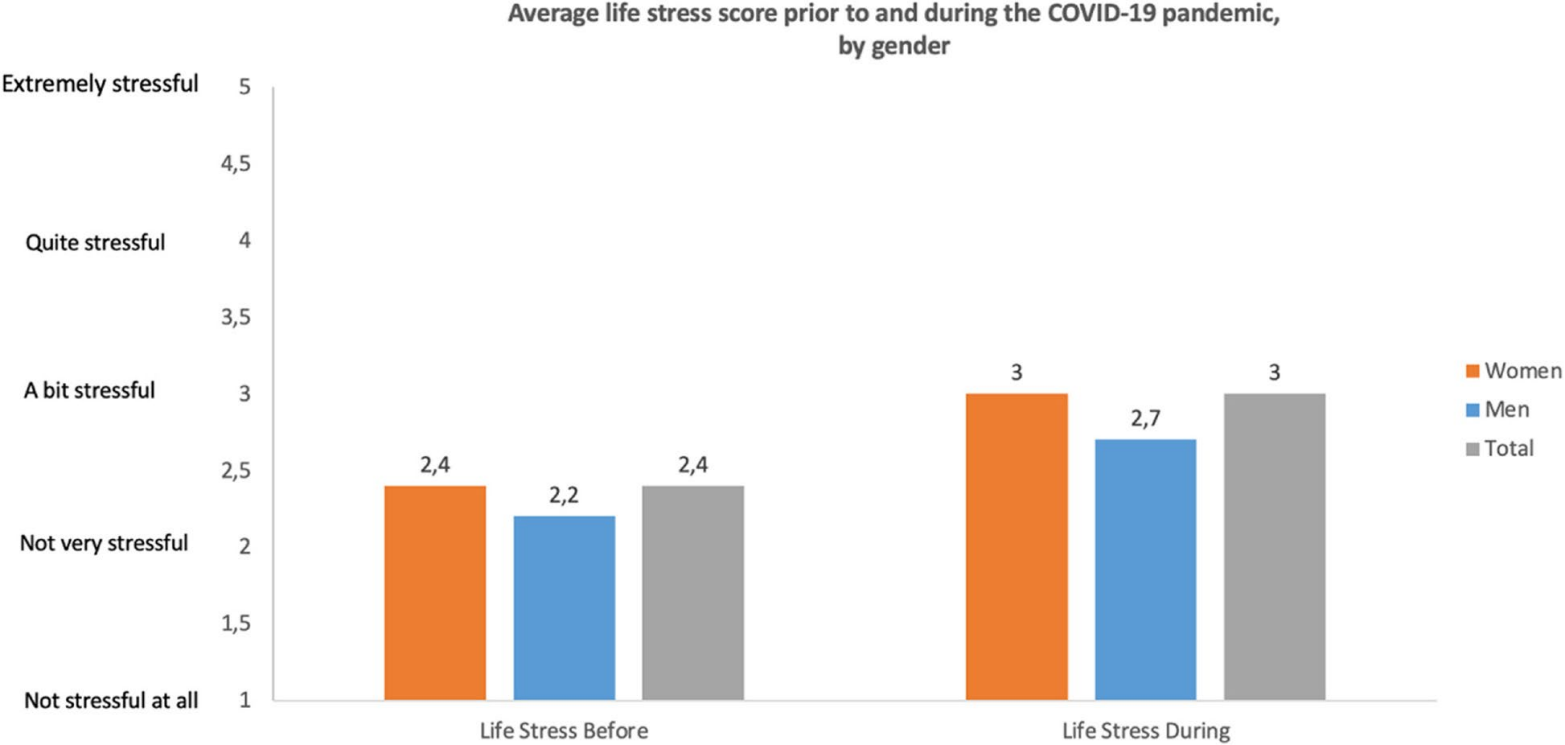
Self-reported life-related stress before and during the COVID-19 pandemic

Gender disparities were noted in the reporting of life stress, with women indicating a significantly higher average increase in work stress during the COVID-19 pandemic in comparison to their stress levels before it (0.62 for women compared to 0.50 for men). Comparable gender disparities were not observed when stratified by profession (Fig. 8).

Both men and women reported higher non-work-related stress levels during COVID-19. Women reported higher levels both before and during the COVID-19 pandemic. Men reported a mean score of 2.2 prior to COVID-19 and 2.7 during COVID-19, which falls between “ "not very stressful" and "a bit stressful". On the other hand, women reported a pre COVID-19 mean score of 2.4, also between "not very stressful" and "a bit stressful”." During COVID-19, they reported a mean of 3.0, indicatin “ "a bit stressful".

**Fig. 9:**
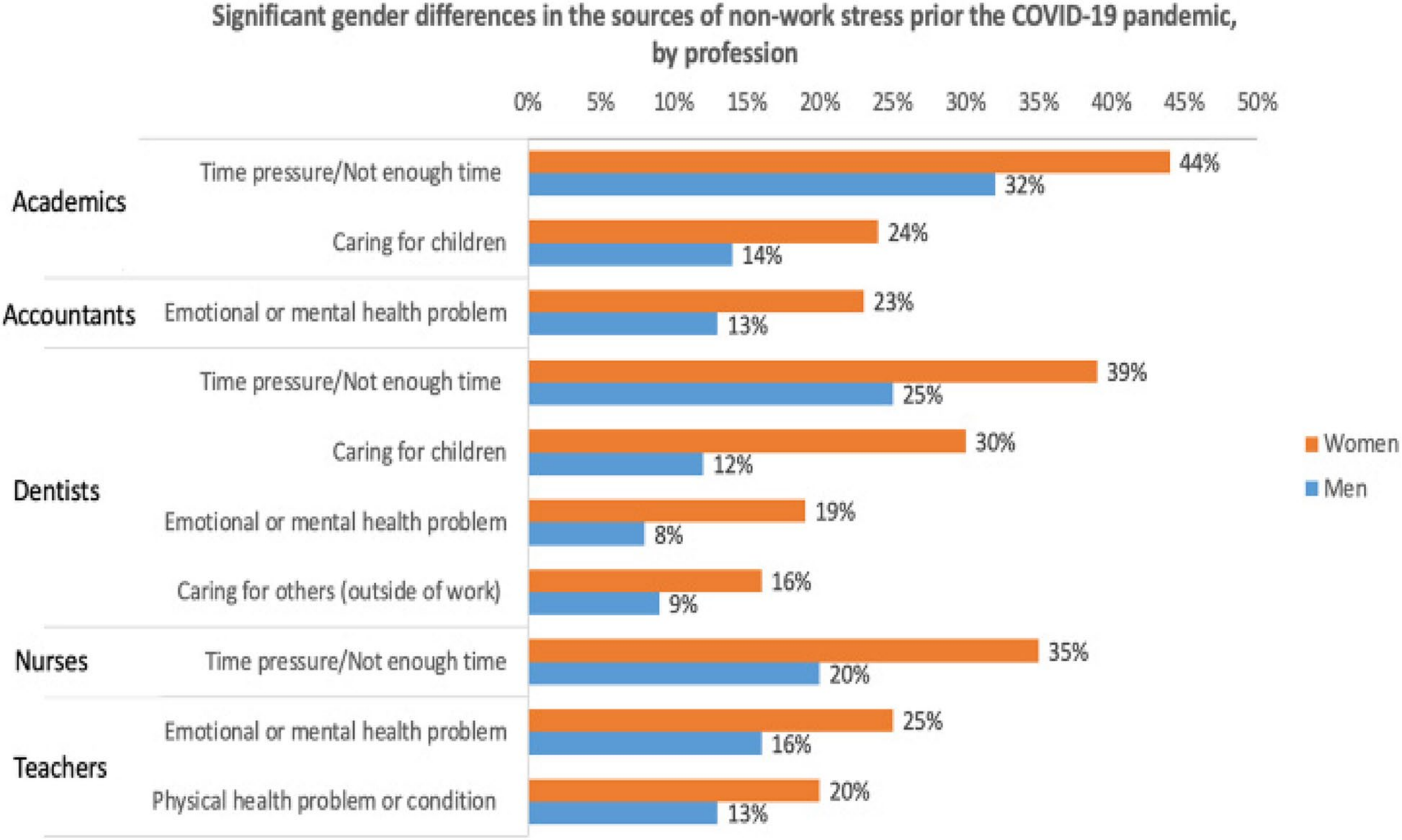
Significant gender differences in the sources of non-work stress prior to the COVID-19 pandemic, by profession

**Fig. 10:**
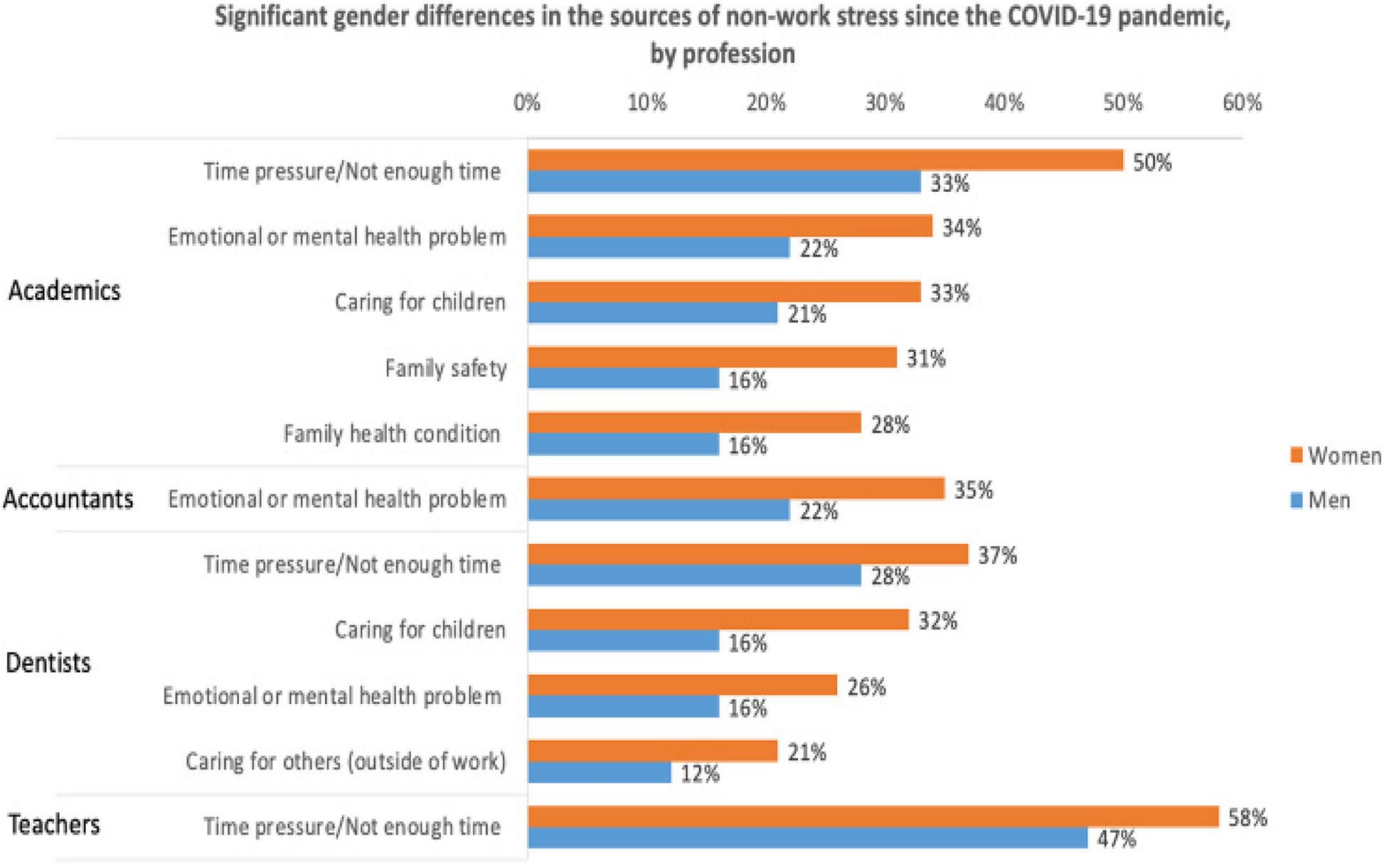
Significant gender differences in the sources of non-work stress since the COVID-19 pandemic, by profession

Prior to COVID-19 work stress was significantly higher than non-work stress for the total population (2.8 vs 2.4), women (2.9 vs 2.4) and men (2.7 vs.2.2). During the pandemic, work stress continued to be significantly higher than non-work stress for the total population (3.4 vs 3.0), women (3.5 vs 3.0) and men (3.2 vs 2.7). All values were higher during COVID-19 compared to pre COVID-19 with women reporting higher values pre and during compared to men (Figs. 9 and 10).

The HPW survey included a comprehensive list of potential sources of non-work stress specific to each profession. Participants were given the option to select multiple sources of stress. Although there were some common options across professions, the lists varied based on the specific occupation.

Time pressure consistently stood out as the primary source of non-work stress across all professions. Caring for children was identified as the main source of non-work stress in the majority of professions. Additionally, physical and mental health conditions were commonly reported as significant sources of non-work stress.

Where significant gender differences were observed, women consistently reported higher proportions than men. Among academia, gender disparities were evident in several aspects. Caring for children was notably different before COVID-19 (24% women vs. 14% men) and during the pandemic (33% women vs. 21% men). Similarly, time pressure exhibited significant gender differences before (44% women vs. 32% men) and during (50% women vs. 33% men) COVID-19. These were the only factors showing gender differences both before and during the pandemic.

Furthermore, the presence of emotional or mental health problems or conditions (34% women vs. 22% men), family safety concerns (31% women vs. 16% men), and family health issues (critical or chronic diseases) (28% women vs. 16% men) displayed significant gender differences during COVID-19 but not before.

Distinct gender disparities were evident among accountants concerning emotional or mental health problems or conditions before (23% women vs. 13% men) and during (35% women vs. 22% men) COVID-19. Although more women made this selection for both time periods, both men and women showed a higher proportion of selection during the pandemic compared to before. Among nurses, the only non-work stressor exhibiting significant gender differences was before COVID-19 where time pressure or not having enough time was selected by 35% of women vs. 20% of men.

In the case of teachers, notable gender disparities emerged in their choices concerning physical health problems or conditions (20% women vs. 13% men) and emotional and mental health issues (25% women vs. 16% men) prior to the onset of COVID-19. A significant gender gap was also evident in terms of time pressure or not enough time, with 58% of women selecting this option compared to 47% of men. In instances where significant gender differences were noted, a higher proportion of women opted for these selections as opposed to men.

Dentists reported significant gender differences in the selection of emotional or mental health problems or conditions before (19% women vs. 8% men) and during (26% women vs. 16% men) the pandemic, as well as in caring for children before (30% women vs. 12% men) and during (32% women vs. 16% men) COVID-19. Additionally, caring for others exhibited gender differences before (16% women vs. 9% men) and during (21% women vs. 12% men) the pandemic, while time pressure or not having enough time showed gender differences both before (39% women vs. 25% men) and during (37% women vs. 28% men) COVID-19.

## Discussion

It is clear from our findings that gender plays a significant role in work and life stress across all professions. Indeed, undertaking an explicit gender-based analysis proved useful as our findings reveal the way work and life stress are uniquely gendered for professional workers. The unique value add we bring to the conversation is the impact of the pandemic on different professionals that are uniquely gendered.

In brief, we found high work and life stress among professional workers compared to other workers, especially among women. Also, the negative impact of the pandemic on work and life stress was found greater among women professional workers. Indeed, the pandemic created a gendered shift with men reporting more stress prior to the pandemic and women reporting more stress during the pandemic, which may be related to the gendered division of labor in the home. This is consistent with existing literature where life stress has been found to have a negative effect on life stress among women but not among men [64].

Our findings add a comparative perspective across several sectors and professions to existing research on how gender impacts stress and anxiety levels [6, 23, 51, 56, 63]. Because women predominate in the caring professions such as nursing and teaching this may explain why time pressure or not having enough time was reported more frequently by women professionals. Teachers and nurses who identify as women reported emotional and mental health issues more often which may reflect a masculine work ethic that tends to surface in these professions [79, 80]. Our data show that in more traditionally masculine professions such as dentistry and academia, caring for children was notably higher for women before and during the pandemic. This demonstrates the importance of understanding if work and life stressors are profession specific or gender specific or interact in some way [73].

Professional workers from all different areas were dissatisfied with their work-life balance during the pandemic Our results reveal the most frequently selected source of work stress was work overload, followed by digital stress, poor work relations, and uncertainty. These findings are in line with studies that show working long hours as a source of work stress among health professionals [20–22] and non-health professionals [23, 24]. We found the primary source of life stress among professional workers was time pressure, caring for children and physical and mental health conditions.. Other research found that as working hours of health professionals increased, work-life balance decreased [44, 45]. While the elusive work-life balance was challenging before the pandemic, the effects of work and life stress on professional worke’s' mental health may be felt for years to come.

These findings have particular relevance for employers and policy makers who are interested in creating sustainable plans for employee recruitment and retention. Focusing efforts on organizational and system level changes could help address the culture change that is needed to prioritize mental health as a sustainable part of work/life balance. Professional workers will continue to experience high work and life stress if addressing work overload and poor mental health are not made a priority. Gender needs to be considered explicitly in these plans and/or interventions. Access to affordable childcare, flexible working schedules, and mental health support would ease the work and life stress experienced by women in the workplace.

### Limitations

Limitations in the data sources include that the data collection for the portion since the pandemic was conducted only for part of the 2020 cycle and the 2021 cycle of CCHS. Thus, COVID-19-related questions did not reflect respondents’ experience of all waves of the pandemic. Moreover, the 2020 and the 2021 cycles of CCHS collected during the COVID-19 pandemic were conducted only by phone interviews, and their collection periods were shortened or interrupted. As a result, there was a significant decrease in the response rates compared to the cycles before the pandemic. Therefore, users are advised to use the CCHS data collected during the COVID-19 pandemic with caution, especially when creating estimates for small sub-populations or when comparing to other CCHS years. As the CCHS is a cross-sectional survey, no causal relationships can be inferred based on the associations found in this analysis.

With respect to the changes from the pandemic, it is important to note thaIhe CCHS data are cross sectional and the HPW data are self-report of differences prior to taking during the second wave of the pandemic. Our analysis was limited to comparing stress levels between men and women, overlooking the intersectionality of diverse gender identities, difficult to undertake given the sample size. Furthermore, the data collection methods, which in the case of the HPW survey relied on crowd-sourcing, might not fully capture the diversity of professions studied. While there were overarching sources of work and life stress, variations in these sources across professions rendered comparisons challenging. Therefore, caution is needed when making broad conclusions based on our observations. To strengthen our findings, future research should validate these results with larger and more representative samples of professional participants.

Building on these findings, future research should further investigate the gender differences that exist between professional workers and how these findings could be utilized to develop appropriate workplace mental health promotion initiatives that are applicable beyond the pandemic.

## Conclusion

The pandemic was multifaceted and had a different impact on various professions depending on the role and structure of their work. Our analysis shows the important role gender plays in life and work stress of professional workers. These findings can help to develop more targeted and appropriate workplace mental health promotion interventions that are applicable to professional workers and are taking gender more fully into consideration. Utilizing a comprehensive approach to implement organization and system level changes will ensure professional workers stay happy and healthy while at work.

## Data Availability

The datasets generated during and/or analyzed during the current study are available from the corresponding author on reasonable request.

## Abbreviations

CCHS: Canadian Community Health Survey
CSP workers: Case study professional workers
HPW: Healthy professional worker

## Footnotes

1 Using NOC 2016, non-professional workers were identified. Non-professional workers included in this analysis were non-professional occupations in business and finance; technical occupations related to natural and applied sciences; technical occupations in health; assisting occupations in support of health services; paraprofessional occupations in legal, social, community and education services; occupations in front-line public protection services; care providers and educational, legal and public protection support occupations; technical occupations in art, culture, recreation and sport; sales and service occupations; trades, transport and equipment operators and related occupations; natural resources, agriculture and related production occupations; and occupations in manufacturing and utilities.

2 The COVID-19 pandemic had major impacts on the data collection operations for CCHS 2020 and CCHS 2021. In 2020, the collection was stopped mid-March, towards the end of the first collection period, and did not resume until September to respect lockdowns and public health. The second, third and fourth quarterly samples were collected during very short collection periods, each of about five weeks, from September to December. Due to the situation of the COVID-19 pandemic, in 2020 and 2021, the in- person interviews were suspended, and the interviews were only conducted over the phone. Typically, the CCHS employs a combination of in-person and telephone interviews. For example, 25% of the 2019 CCHS was collected by in-person interview and 75% by telephone interviews [75], Also, the collection of the 2021 CCHS was interrupted in the month of May for the Canadian Census of Population. It was initially planned to have six two-month collection periods. Due to operational constraints related to the 2021 Census, the collection period originally scheduled from May 1st to June 30th was rescheduled to be collected between June 1st and September 5th. The final two collection periods also have been rescheduled to be collected September 1st to November 14th and November 15th to February 7th. These changes were made in order to meet targeted response rates.

3 Note. 756 education workers selected their role as teacher. Those who opted for “other” role and specified roles similar to teaching were included in the teacher group.
